# PCK1 Downregulation Promotes TXNRD1 Expression and Hepatoma Cell Growth via the Nrf2/Keap1 Pathway

**DOI:** 10.3389/fonc.2018.00611

**Published:** 2018-12-17

**Authors:** Lin Tuo, Jin Xiang, Xuanming Pan, Qingzhu Gao, Guiji Zhang, Yi Yang, Li Liang, Jie Xia, Kai Wang, Ni Tang

**Affiliations:** ^1^Key Laboratory of Molecular Biology for Infectious Diseases (Ministry of Education), Department of Infectious Diseases, Institute for Viral Hepatitis, The Second Affiliated Hospital, Chongqing Medical University, Chongqing, China; ^2^Department of Pathogenic Biology, Basic Medical College, Chongqing Medical University, Chongqing, China

**Keywords:** phosphoenolpyruvate carboxylase kinase 1, thioredoxin reductase 1, hepatocellular carcinoma, reactive oxygen species, nuclear factor erythroid 2-related factor 2

## Abstract

Gluconeogenesis, generates glucose from small carbohydrate substrates, and drives the metabolic flux in parallel but opposite to glycolysis. The cytoplasmic isoform of phosphoenolpyruvate carboxykinase (PCK1 or PEPCK-C), a rate-limiting enzyme in gluconeogenesis, initiates the gluconeogenesis process and is reportedly dysregulated in multiple types of cancer. Gluconeogenesis mainly occurs in the liver during fasting, and previous studies have demonstrated that PCK1 acts as a tumor suppressor in hepatocellular carcinoma (HCC); however, the role of PCK1 in cancer progression remains incompletely understood. In the current study, we found that PCK1 expression was decreased in HCC as compared to adjacent normal liver tissues, and low PCK1 expression correlated with poor patient prognosis. Furthermore, overexpression of PCK1 suppressed reactive oxygen species (ROS) production and nuclear translocation of Nrf2 in hepatoma cells. In addition, thioredoxin reductase 1 (TXNRD1), an antioxidant enzyme regulated by the Nrf2/Keap1 pathway, was downregulated upon overexpression of PCK1 in HCC cell lines. Furthermore, we verified this axis using nude mouse xenograft model. Finally, we found that auranofin, a TXNRD1 inhibitor, enhanced the sensitivity of PCK1-knockout hepatoma cells to sorafenib-induced apoptosis. Taken together, our findings suggest that PCK1 deficiency promotes hepatoma cell proliferation via the induction of oxidative stress and the activation of transcription factor Nrf2, and that targeting the TXNRD1 antioxidant pathway sensitizes PCK1-knockout hepatoma cells to sorafenib treatment *in vitro*.

## Introduction

Hepatocellular carcinoma (HCC), the fifth most common cancer worldwide and the third cause of cancer-related mortality (1), is liable to developing malignant and drug-resistant tumors, with high risk of recurrence. A thorough understanding of the molecular pathogenesis of HCC and novel therapeutic strategies are highly needed. Metabolic reprogramming is a hallmark of cancer (2). In normal cells, energy is obtained primarily through mitochondrial oxidative phosphorylation. However, tumor cells undergo glycolysis even in the presence of oxygen, known as the “Warburg effect,” which supports tumor growth by accumulating glycolytic intermediates for anabolic biosynthesis (3).

The changed glucose metabolism endows tumor cells with metabolic flexibility for biosynthesis requirements. The dysregulation of gluconeogenesis and glycolysis causes a metabolic reprogramming that might be essential for supporting the growth, proliferation, and survival of tumor cells. Meanwhile, emerging evidence is linking altered expression of gluconeogenic enzymes, in particular, phosphoenolpyruvate carboxykinase (PCK, also known as PEPCK, EC number 4.1.1.32), with tumorigenesis (4). PCK1, as the first rate-limiting enzyme of gluconeogenesis, catalyzes the conversion of oxaloacetate (OAA) to phosphoenolpyruvate (PEP), and its dysfunction has been related to diabetes, obesity, insulin resistance, fatty liver, and other metabolic diseases (5, 6). Elevated expression of PCK1 has been reported to benefit tumor growth in certain cancers by maintaining anabolic metabolism (7, 8). In contrast, in HCC, PCK1 is downregulated (9), and a functional study indicated that forced PCK1 expression retards hepatoma cell proliferation (10), however, the underlying mechanism remains to be clarified.

Reactive oxygen species (ROS), such as hydrogen peroxide (H_2_O_2_), hydroxyl radical (HO•), and superoxide anion (${\text{O}}_{2}^{-}$), are formed by the partial reduction of oxygen and are generated as normal byproducts of numerous cellular processes (11). Under physiological conditions, ROS are maintained at low levels, and act as signaling molecules to activate cell proliferation and survival pathways (12). However, excessive ROS production induces oxidative stress, causing cell death or apoptosis, and plays an important role in tumor initiation and progression (13). The nuclear factor erythroid 2-related factor 2 (Nrf2)/Kelch-like ECH-associated protein 1 (Keap1) signaling pathway is one of the crucial defense systems against oxidative stress in tumor tissues and cells (14). ROS production induces Nrf2 translocation from the cytoplasm to nucleus, where it forms heterodimers with small MAF proteins and binds the antioxidant response element (ARE) in the promoters of target genes to activate their transcription to protect against oxidative stress.

Thioredoxin reductase 1 (TXNRD1), an antioxidant enzyme regulated by Nrf2, catalyzes reduction of the active site disulfide of thioredoxin 1 (Trx1), and several other substrates and plays a key role in antioxidant defense, redox regulation, cell proliferation, and cell signaling events (15). TXNRD1 is highly expressed in many cancers, and has been linked with tumor development (3, 13, 16). Recent studies aimed to suppress TXNRD1 as a novel therapeutic target in the treatment of cancers by using various molecules, such as microRNA-124 (17), auranofin (18), and gambogic acid (19). Mechanically, the Trx1 system senses and responds to ROS generated by cellular respiration, metabolism, and immune response, and then regulates the redox status, function, and activity of its target signal pathway (20). However, it is not clear whether PCK1 modulates the expression of TXNRD1 by influencing the ROS levels.

In this study, we aimed to investigate the role of PCK1 in HCC development *in vitro* and *in vivo*. We explored whether PCK1 plays a role in oxidative stress and Nrf2/Keap1 pathway. We also investigated the antitumor effect of auranofin combined with sorafenib in PCK1 knockout hepatoma cells *in vitro*. Our results implicate that PCK1 acts as a tumor suppressor in HCC and combination therapy of sorafenib and TXNRD1 inhibitors may be a novel strategy for HCC treatment.

## Materials and Methods

### Patient Samples

Surgically resected samples of HCC tumor tissues and adjacent non-tumorous tissues were obtained from 28 patients at the Department of Hepatobiliary Surgery of the Second Affiliated Hospital of Chongqing Medical University (Chongqing, China). Twenty-eight freshly collected paired HCC tissues were frozen immediately and stored in liquid nitrogen for further research. The patients enrolled in this study did not receive preoperative radiotherapy or chemotherapy.

### Cell Cultures and Drug Treatment

The human hepatoma cell line PLC/PRF/5 was obtained from the American Type Culture Collection (Manassas, VA, USA). Huh7 cells were obtained from the Cell Bank of the Chinese Academy of Sciences (Shanghai, China). All cell lines used in this study were recently authenticated by short tandem repeat profiling (Beijing Microread Gene Technology Co., China). Cells were cultured in Dulbecco's modified Eagle's medium (Hyclone, Logan, UT, USA) containing 10% fetal bovine serum (Gibco, Rockville, MD, USA), 100 IU penicillin, and 100 mg/ml streptomycin at 37°C in a humidified atmosphere containing 5% CO_2_.

For cell proliferation assay, immunoblotting, and apoptosis-inducing factor studies, cells were plated in triplicate and incubated with sorafenib (5 μmol/L, Cat# S7397; Selleck, Houston, TX, USA), auranofin (0.6 μmol/L, Cat# HY-B1123; MedChem Express, Princeton, NJ, USA), or both sorafenib and auranofin for 72 h and then collected for analysis.

### Plasmid Constructs and Adenovirus Production

The full-length cDNA of PCK1 (coding sequence of NM_002591) was subcloned from plasmid pOTB7-PCK1 (Cat# FL07339; GeneCopoeia, Guangzhou, China) and inserted into the shuttle vector pAdTrack-TO4 (kindly gifted by Dr. Tong-Chuan He, University of Chicago, USA). Primers are shown in Table S1. Adenoviral recombinant AdPCK1 was generated using the AdEasy system, as described previously (7). Green fluorescent protein-expressing analogous adenovirus (AdGFP) was used as a control.

### RNA Extraction and Reverse Transcription Real-Time (RT-q)PCR

Huh7 cells were infected with AdPCK1 or AdGFP for 36 h. Total RNA was extracted from cultured cells or fresh tissue samples using TRIzol reagent (Invitrogen, Rockville, MD, USA), following the manufacturer's instructions. The RNA was reverse transcribed using Moloney murine leukemia virus reverse transcriptase (Promega) and random hexamers (Promega, Madison, WI, USA). SYBR Green was used for quantitative PCR in a CFX Real-Time PCR Detection System (Bio-Rad, Hercules, CA, USA). The relative mRNA levels were calculated using the 2^−ΔΔ*Ct*^ method. Each in triplicate. All primers are shown in Table S1. The actin beta gene (*ACTB*) was used as reference gene for normalization in both cells and tissues.

### Western Blotting Analysis

Cells and tissue samples were harvested and treated with lysis buffer (Beyotime, Shanghai, China) containing 1 mM phenylmethylsulfonyl fluoride (Beyotime). Protein concentrations were determined using a BCA protein assay kit (Beyotime). Comparable amounts of proteins were subjected to 10% sodium dodecyl sulfate polyacrylamide gel electrophoresis and electrotransferred to polyvinylidene difluoride membranes (Millipore, Billerica, MA, USA). The membranes were probed with antibodies against PCK1 (1:1000; Cat# BS6870; Bioworld, Atlanta, GA, USA), TXNRD1 (1:500; Cat# 15140; Cell Signaling), or NRF2 (1:1000; Cat# 12721; Cell Signaling). Secondary antibodies conjugated to horseradish peroxidase were purchased from Abcam (Cambridge, MA, USA). Protein bands were visualized using Super Signal West Pico Chemiluminescent Substrate Kit (Millipore) and quantified using ImageJ software (National Institutes of Health, Bethesda, MA, USA, http://imagej.nih.gov/ij/). GAPDH (Cat# AF0006; Beyotime), β-actin (Cat# BL005B; Biosharp), laminB1 (Cat# AP6001; Bioworld), and β-tubulin (Cat# AP0064; Bioworld) were used as normalization controls. Nuclear and cytoplasmic extracts of cells were collected using a Nuclear and Cytoplasmic Protein Extraction Kit (Beyotime). All experiments were repeated three times independently.

### Immunohistochemistry

Paraffin-embedded liver tissue samples were obtained from the same 28 patients described above, and then sliced and deparaffinized according to standard procedures. The slides were incubated with anti-PCK1 antibody at 4°C overnight. After washing with PBS, the sections were incubated with secondary anti-rabbit IgG (ZSGB-BIO, Beijing, China) and stained with 3,3'-diaminobenzidine (ZSGB-BIO). For immunohistochemical assessment, the staining images of 3 pairs of HCC and adjacent non-tumor tissues were quantified using integrated optical density (IOD) by Image-Pro Plus 6.0 software.

### RNA-Sequencing (RNA-Seq) and Expression Analysis

For RNA sequencing, Huh7 cells were infected with AdGFP (*n* = 3) or AdPCK1 (*n* = 3) for 36 h. Total RNA was extracted using Trizol reagent (Invitrogen), and quantified using a NanoDrop ND-1000 spectrophotometer (Thermo Fisher Scientific, Wilmington, DE, USA) and a Bioanalyzer 2,200 (Agilent Technologies, CA, USA). Then, 5 μg RNA with a RNA Integrity Number (RIN) above 8.0 was used for cDNA library construction. RNA-seq and bioinformatic data analysis were performed by Shanghai Novel Bio Ltd. Briefly, strand-specific RNA-seq libraries were prepared using the Total RNA-seq (H/M/R) Library Prep Kit (Vazyme Biotech, Nanjing, China) and were sequenced on an Ion Torrent Proton Sequencer (Life Technologies, Carlsbad, CA, USA) according to Ion PI Sequencing 200 Kit v2.0 (Life Technologies). Raw reads in FASTQ format were subjected to quality control using FastQC (http://www.bioinformatics.babraham.ac.uk/projects/fastqc/). RNA-seq reads were aligned to the reference genome using Bowtie (http://bowtie-bio.sourceforge.net). Uniquely mapped reads were used for further analysis. Gene expression levels are expressed as RPKM (reads per kilobase per million reads) and differences in gene expression were calculated with rSeq (http://www-personal.umich.edu/~jianghui/rseq).

The RNA-seq data generated in this study have been deposited in the National Center for Biotechnology Information (NCBI) Gene Expression Omnibus database (GEO, http://www.ncbi.nlm.nih.gov/geo) under accession number GSE117822.

### CRISPR/Cas9-Mediated Knockout of PCK1

The CRISPR/Cas9 plasmids lentiCRISPR v2, pMD2.G, and psPAX2 were kindly provided by Prof. Ding Xue from the School of Life Sciences, Tsinghua University (Beijing, China). Single-guide RNAs (sgRNAs) targeting human PCK1 were designed by using the E-CRISP online tool (http://www.e-crisp.org/E-CRISP/designcrispr.html). The DNA oligonucleotides were annealed and cloned into the lentiCRISPR v2 vector digested with *BsmB*I (Thermo Fisher Scientific, Waltham, MA, USA). HEK293T cells were co-transfected with PCK1 sgRNAs or empty lentiCRISPR v2 as a control, envelop plasmid pMD2.G, and packaging plasmid psPAX2 using Lipofectamine 2,000 (Thermo Fisher Scientific, Waltham, MA, USA), following the manufacturer's protocol. Medium containing lentiviral particles and 5 μg/mL polybrene were used to infect PLC/PRF/5 cells. Two days post infection, cells were selected in medium containing 2 μg/mL puromycin. Then, the cells were trypsinized from the plates and seeded into a 96-well plate (1 cell/well, theoretically). The culture medium was replaced with fresh, puromycin-containing medium every 2 days until drug-resistant single colonies were identified by western blot analysis. For genotyping, clonal cell genomic DNA was extracted with Genomic DNA Purification Kit (Genloci Biotechnologies Inc, Jiangsu, China) according to the manufacturer's instructions. The genomic region flanking the gRNA target site was amplified by touch down PCR, and then subsequently cloned into the pMD19-T TA cloning vector (Takara, Kyoto, Japan) and sequenced (Figure S1). PCK1 knockout efficiency was confirmed by western blotting. PCK1-knockout cells and control cells were defined as KO 1/KO 2 cells and parental cells, respectively. All primers are shown in Table S1.

### Cell Proliferation Assay

The IncuCyte ZOOM Live-Cell Imaging system (Essen BioScience, Ann Arbor, Michigan, USA) was used for monitoring cell proliferation. Cells were seeded in 96-well plates at 1 × 10^3^ cells/well and were cultured for 5 days. The plate was scanned and phase-contrast images were acquired in real time every 24 h post treatment, and quantified time-lapse curves were generated by IncuCyte ZOOM software. The medium was replaced with fresh medium containing the respective reagents at 3-day intervals.

### Cell Apoptosis Assay

Cells were seeded at a density of 1.0 × 10^6^ cells/mL and treated with different reagents. After washing twice with PBS, the cells detached with trypsin, collected by centrifugation and incubated with reagents from the Annexin-V-FITC Apoptosis Detection Kit (Biotool, Shanghai, China) according to the manufacturer's protocol. Then the cells were analyzed by FACS Vantage SE flow cytometer (BD Biosciences, San Jose, CA, USA). The experiment was repeated three times independently.

### Measurement of NADP/NADPH

NADPH and NADP^+^ levels were measured using a NADP/NADPH Assay Kit (ab65349, Abcam, Cambridge, UK) according to the manufacturer's instructions. Cell lysates were extracted with provided buffer, then mixed with NADP cycling solution and with the NADPH developer. The absorbances of NADP and NADPH were measured at OD450 nm, and the concentrations were calculated by comparison with the standard curve.

### ROS Assay

Huh7 cells were infected with AdGFP or AdPCK1 in 6-well plates. Frozen tumors were sectioned at 10 μm. Tissues sections, infected Huh7, KO, and parental cells were incubated with 5μM of CellROX Deep Red Reagent (Thermo Fisher Scientific) for 30 min. The tissues and cells were washed 3 times with PBS and then, the nuclei were stained with 1 μg/mL DAPI (Sigma, St. Louis, MO, USA) for 3 min. The fixed tissues and cells were imaged under a confocal laser-scanning microscope (Leica, Heidelberg, Germany). The proportions of ROS-positive to total tissues or cells were determined by fluorescence microscopy in five random fields (magnification, 50× or 200×) in three wells.

### Xenograft Tumor Formation Assay

For the subcutaneous xenograft tumor model, 18 BALB/c nude mice (male, 5–6 weeks of age) were randomly divided into Mock, AdGFP and AdPCK1 groups (6 mice per group). MHCC-97H cells were mock-infected or infected with AdGFP, AdPCK1 for 24 h, collected and subcutaneously injected into the flanks of nude mice (1 × 10^5^ cells/injection). Tumor volumes were measured at 4-day intervals and calculated as length [cm] × (square of width [cm])/2. Mice were sacrificed after 4 weeks, and tumor tissues were removed for assessment. The protocol of animal experiments was approved by the Institutional Animal Care and Use Committee at Chongqing Medical University, and adhere to National Regulations for the administration of laboratory animal.

### Statistical Analysis

Data are presented as the mean ± standard deviation (SD). Survival rates were calculated using the Kaplan-Meier method and statistical significance was evaluated using the log-rank test. Statistical significance was determined using Student's *t*-test when comparing 2 groups or one-way analysis of variance (ANOVA) when comparing more than 2 groups. Statistical analysis was conducted using GraphPad Prism 7.0 software (La Jolla, CA, USA). *P*-values < 0.05 were considered statistically significant.

## Results

### PCK1 Expression Is Downregulated in HCC

**Figure 1:**
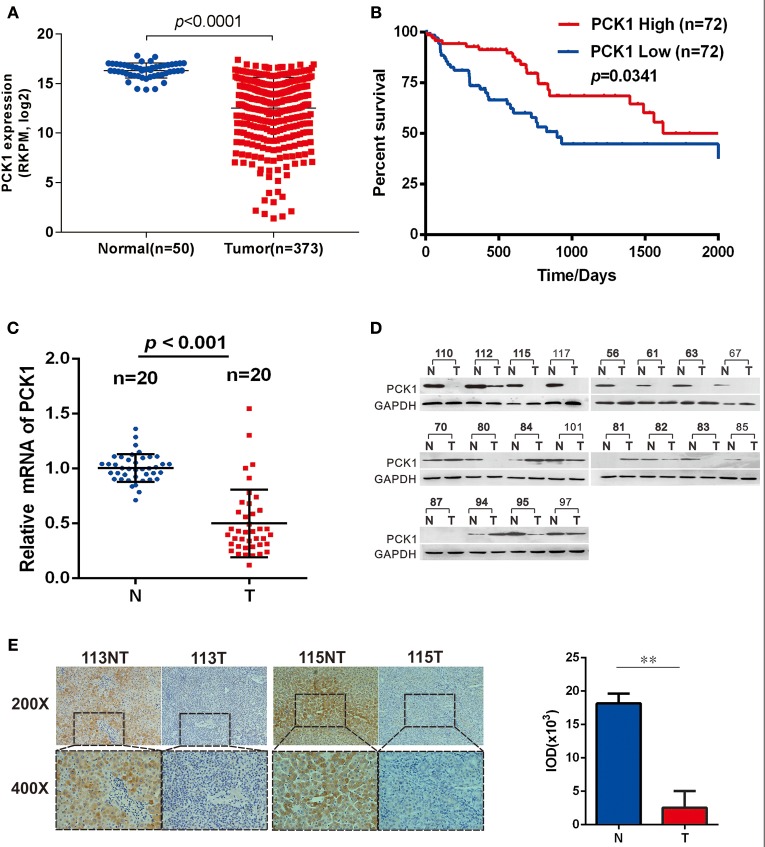
PCK1 is generally decreased in hepatocellular carcinoma (HCC) tissues and correlates with poorer patient prognosis. **(A)** Expression of PCK1 in patients with HCC in the TCGA Liver Hepatocellular Carcinoma (LIHC) dataset. **(B)** Kaplan Meier survival curve based on data for 142 HCC patients in the TCGA dataset, divided into two groups by PCK1 expression levels in tumors. N represents paratumor tissues. T represents tumor tissues. **(C)** RT-qPCR analysis of PCK1 expression in 20 paired HCC and adjacent non-tumorous tissues (*P* < 0.001). **(D)** PCK1 protein levels in 20 paired primary HCC tissues and adjacent non-tumor tissues determined by western blotting. β-actin was used as a loading control. **(E)** Representative immunohistochemistry (IHC) images of PCK1 in HCC and adjacent non-tumor tissues. Immunostaining intensity was measured using ImagePro Plus 6.0 software. (^**^P < 0.01); magnification: 400×, 200×. Statistical analysis of PCK1 protein levels from 4 pairs of HCC and adjacent non-tumor tissues as determined by IHC staining. The cropped blots are used in the figure and full length blots are presented in Figure S2.

To clarify the function of PCK1 during the carcinogenesis of HCC, we first analyzed PCK1 expression levels in an independent cohort of 373 HCC samples (including 50 paired HCC tissues and para-tumor tissues) from The Cancer Genome Atlas (TCGA) database. The data showed that PCK1 expression was significantly lower in tumor tissues than in normal liver tissues (Figure 1A). In addition, we interpreted 72 cases as “PCK1 High” or “PCK1 Low” depending on the PCK1 expression level. Survival curve analysis demonstrated that patients with low expression of PCK1 had lower overall survival than patients with high expression of PCK1 (median survival, 835 vs. 1,630 days; Figure 1B). We further detected the PCK1 expression levels in 20 pairs of HCC and adjacent non-cancerous tissues from patients at the second affiliated Hospital of Chongqing Medical University. PCK1 mRNA levels were downregulated in HCC tissues compared to adjacent normal tissues (*P* < 0.01, Figure 1C). In addition, PCK1 protein levels were markedly lower in 16 out of the 20 HCC tissues (85%, Figure 1D). Immunohistochemical staining showed that PCK1 expression levels were significantly lower in HCC than in para-tumor tissues (*P* < 0.01, Figure 1E). Collectively, these data indicated that PCK1 is generally downregulated in HCC tissues, which is correlated with poorer prognosis.

### PCK1 Represses TXNRD1 Expression in Hepatoma Cells

**Figure 2:**
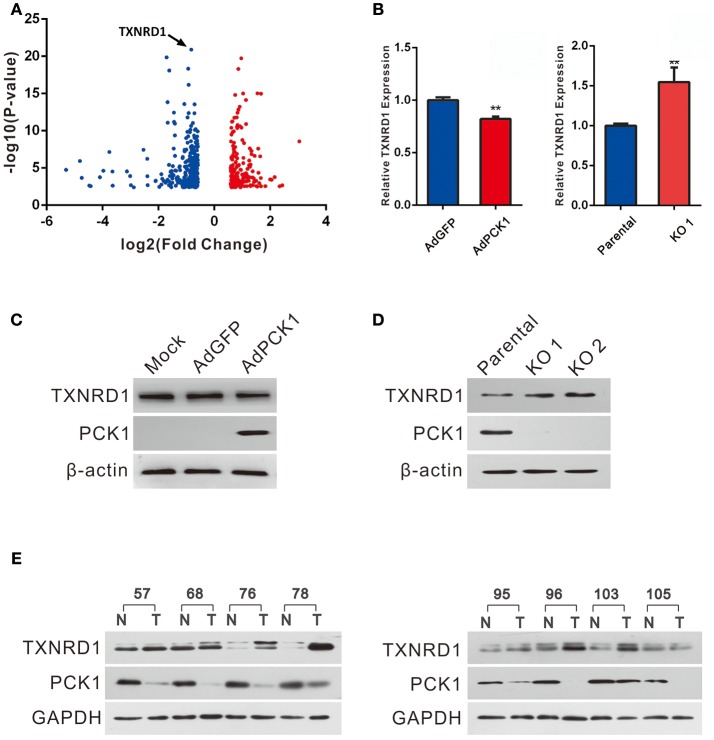
PCK1 represses TXNRD1 expression in hepatoma cells and hepatic tissues**. (A)** Volcano plot of RNA-seq data from Huh7 cells infected with adenoviruses expressing PCK1 (AdPCK1) vs. Huh7 cells infected vector control (AdGFP). In total, 498 genes were differentially expressed (fold change >1.5 or < 0.667; FDR < 0.05). TXNRD1 is downregulated in PCK1-overexpressing hepatoma cells. **(B)** RT-qPCR analysis of mRNA levels of TXNRD1 in Huh7 cells infected with AdPCK1 or AdGFP. Total mRNA was isolated at 36 h after infection. Elevated mRNA expression of TXNRD1 in PCK1-KO (endogenous PCK1 was knocked out in PLC/PRF/5 cells by CRISPR-Cas9) compared to parental cells. Data are shown as the mean ± SD (*n* = 3 independent experiments; **P < 0.01 by Student's *t*-test). **(C)** Western blot analysis of TXNRD1 in Huh7 cells infected with AdPCK1 or AdGFP. **(D)** Protein levels of TXNRD1 were detected by western blot analysis in PCK1-KO cells and parental cells. **(E)** PCK1 and TXNRD1 protein levels in eight cases of paired primary hepatocellular carcinoma tissues and adjacent non-tumor tissues. GAPDH was used as a loading control. The cropped blots are used in the figure and full length blots are presented in Figure S3.

Huh7 PCK1-overexpressing as well as PLC/PRF/5 PCK1-knockout cell lines were used to investigate the potential function of PCK1 in hepatoma cell proliferation. RNA-seq analysis demonstrated that the expression of 498 genes was significantly changed in AdPCK1- vs. AdGFP-infected Huh7 cells. Among these differentially expressed genes, *TXNRD1*, encoding a critical antioxidant enzyme, was remarkably downregulated (*P* < 0.05, Figure 2A and Table S2). Suppression of both TXNRD1 mRNA and protein expression was verified in PCK1-overexpressing Huh7 cells by RT-qPCR and immunoblot analysis (Figures 2B,C). In PCK1-KO PLC/PRF/5 cells, both TXNRD1 mRNA and protein levels were enhanced (Figures 2B,D). In addition, we examined the protein levels of PCK1 and TXNRD1 in eight cases of paired HCC and adjacent non-cancerous tissues. Downregulation of PCK1 (8/8) and upregulation of TXNRD1 (6/8) in HCC tissues were observed by immunoblotting (Figure 2E). These results indicated that PCK1 significantly downregulated TXNRD1 expression in hepatoma cells, and that the expression levels of PCK1 were negatively correlated with TXNRD1 expression in HCC tissues.

### PCK1 Suppresses Oxidative Stress in Hepatoma Cells

**Figure 3:**
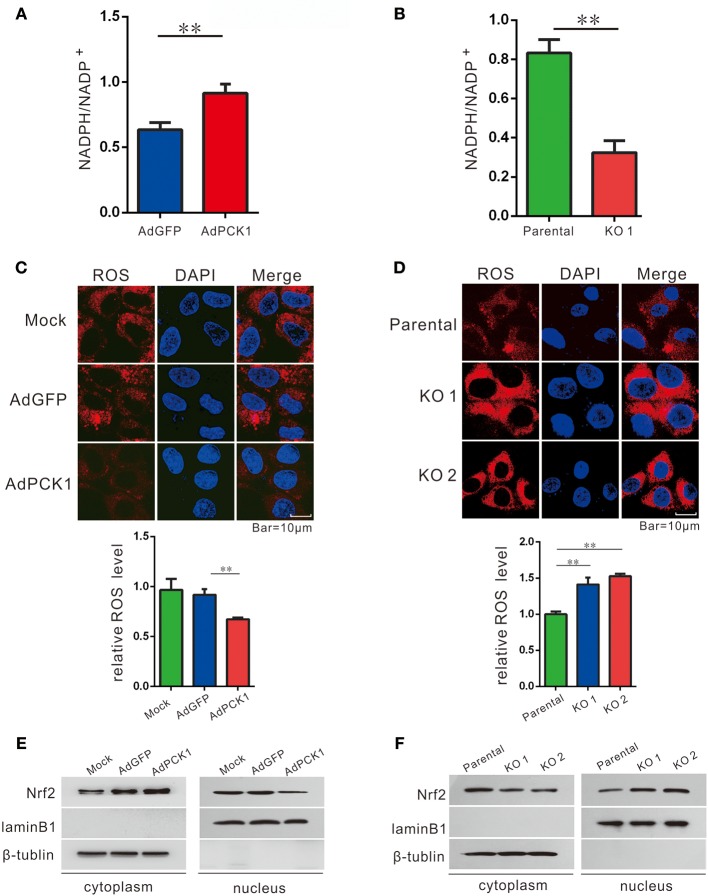
PCK1 suppresses oxidative stress in liver cancer cells. Relative ratios of NADPH to total NADP in PCK1-overexpressing cells **(A)**, and PCK1-KO cells **(B)** were measured by using an NADP/NADPH quantification kit (Abcam). Fluorescent labeling of ROS (red) in the cytoplasm, nuclei were stained with DAPI (blue). Scale bar = 10 μm. ROS levels in PCK1-overexpressing cells **(C)**, and PCK1-KO cells **(D)** were detected, and the relative fluorescence intensities were determined after normalization to mock-infected or parental cells. Data are represented as the mean ± SD (*n* = 3, technical replicates; **P < 0.01 by Student's *t*-test). **(E)** Protein expression of Nrf2. Huh7 cells were treated as described in Figure 2. Two days post infection, nuclear and cytoplasmic fractions were obtained and analyzed by western blotting. LaminB1 and β-tubulin were used as internal loading controls. **(F)** Nuclear accumulation of Nrf2 in PCK1-KO cells. Nuclear and cytoplasmic fractions were obtained and analyzed by western blotting. LaminB1 and β-tubulin were used as internal loading controls for western blotting. The cropped blots are used in the figure and full length blots are presented in Figure S4.

Previous studies have indicated TXNRD1 plays an important role in redox homoeostasis, and its expression could be induced by ROS production. (11, 13, 21) Furthermore, NADPH/NADP^+^, which is necessary for redox balance, can be regulated by the pentose phosphate pathway (PPP) (22). Thus, we examined NADPH/NADP^+^ ratio and ROS levels in hepatoma cells upon PCK1 expression. As expected, the ratio of NADPH/NADP^+^ was increased in PCK1-overexpressing hepatoma cells (Figure 3A), while decreased in PCK1-KO PLC/PRF/5 cells (Figure 3B), compared to control cells. Specifically, we found that the ROS production was significantly reduced in PCK1-overexpressing Huh7 cells, while compared to parental cells, higher production of ROS was detected in PCK1-KO PLC/PRF/5 cells (Figures 3C,D). As Nrf2 is a key transcription factor in triggering ROS-induced gene expression, (13, 23) the regulatory effect of PCK1 on Nrf2 cellular distribution was also investigated. Immunoblot results revealed a remarkably increased cytoplasmic but decreased nuclear level of Nrf2 in PCK1-overexpressing Huh7 cells (Figure 3E). In contrast, the nuclear levels of Nrf2 were elevated in PCK1-KO cells compared to parental cells (Figure 3F). These results suggested that overexpression of PCK1 suppresses both ROS production and the nuclear translocation of Nrf2.

### PCK1 Exerts Antitumor Effect by Inhibiting ROS Level and TXNRD1expression *in vivo*

**Figure 4:**
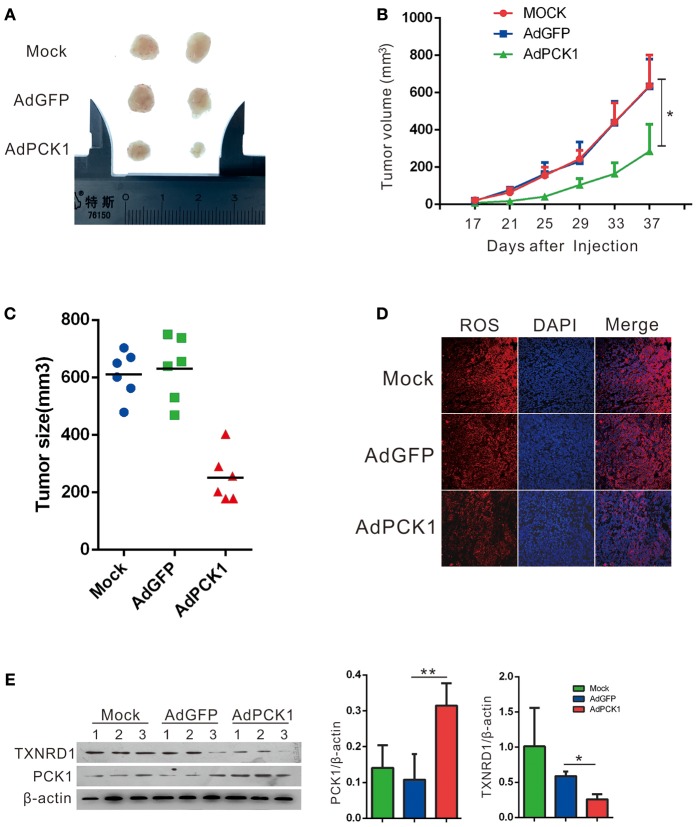
PCK1 inhibits tumor growth and oxidative stress in xenograft tumors. MHCC-97H cells were infected with AdGFP or AdPCK1 or mock-infected for 24 h, then subcutaneous injected into nude mice (*n* = 6). **(A)** Representative image of tumor in all groups (presented two tumors each group). **(B)** The growth curve of subcutaneous xenograft tumor. Tumor volumes were measured every 4 days and calculated as length × (square of width)/2 (repeated-measures analysis of variance, *P < 0.05 compared to AdGFP group, error bars represent mean ± SD, *n* = 6). **(C)** Tumor size of subcutaneous xenografts after dissected. **(D)** ROS monitored by immunofluorescent labeling (red) and nuclei by DAPI (blue) staining in xenografts. Scale bar = 200 μm. **(E)** Expression of PCK1 and TXNRD1 checked by western blot assay in xenograft tumor samples. Semi-quantitative densitometry graphs of immunoblots (arbitrary densitometry units) for PCK1 and TXNRD1 levels normalized to β-actin expression are shown. (*P < 0.05, **P < 0.01, AdPCK1 group vs. AdGFP group). The cropped blots are used in the figure and full length blots are presented in Figure S5.

The antitumor effect *in vivo* was evaluated in MHCC-97H xenograft tumor in nude mice. All mice developed xenograft tumors at the injection site. Twenty days after injection, we noticed that the tumors were much smaller in the PCK1-overexpression group than in the mock and the empty vector group (Figure 4A). As shown in Figure 4B, overexpression of PCK1 significantly reduced the growth rate of MHCC-97H tumor growth compared with the mock and GFP control group by measuring tumor volume every 4 days (^*^P < 0.05, AdPCK1 group vs. AdGFP group, Figure 4B). In addition, the average tumor size was smaller in the PCK1-overexpression group than that in the control group (Figure 4C). We also found that ROS production was significantly reduced in PCK1-overexpression group (Figure 4D). Furthermore, western-blot analysis was used to analyze the expression levels of PCK1 and TXNRD1 in the xenograft tumor tissues. Immunoblotting results revealed higher expression of PCK1 and lower expression of TXNRD1 in tumor tissues derived from the PCK1-overexpressing hepatoma cells than in GFP control group (Figure 4E). Together, these results suggested that PCK1 could inhibit hepatoma cell growth *in vivo* by decreasing ROS level and TXNRD1 expression.

### Auranofin Enhances the Sensitivity to Apoptosis Induced by Sorafenib Treatment via Inhibition of TXNRD1 in PCK1-KO Cells *in vitro*

**Figure 5:**
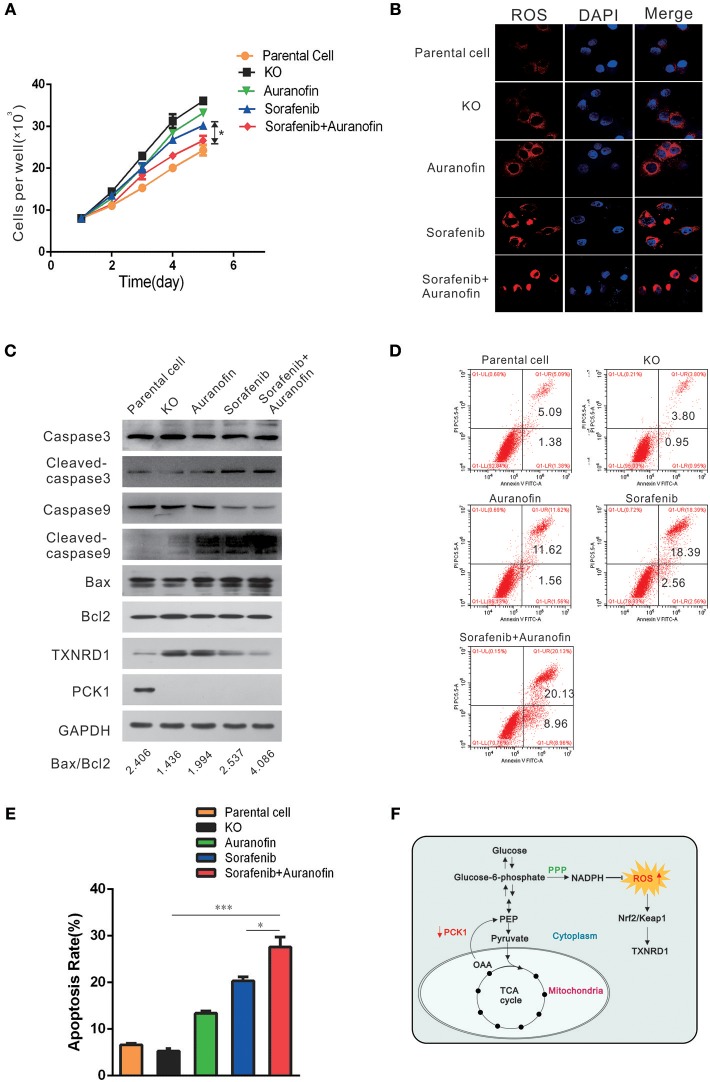
Auranofin enhances the sensitivity to sorafenib-induced apoptosis via inhibition of TXNRD1 in PCK1-KO cells *in vitro*. **(A)** Cell proliferation curves. PCK1-KO hepatoma cells were treated with 0.6 μmol/L auranofin or 5 μmol/L sorafenib alone or in combination. Cells were plated into 96-well plates at 1 × 10^3^ cells/well and were counted every 24 h in triplicate. Data are presented as the mean ± SD; *P < 0.05 vs. Sorafenib alone. **(B)** ROS were monitored by fluorescent labeling (red) and nuclei were stained by DAPI (blue) in cells treated with sorafenib and auranofin alone or in combination. Scale bar = 10 μm. **(C)** Protein expression of Caspase3/9, Cleaved caspase3/9, Bax, Bcl2, TXNRD1, and PCK1. PCK1-KO hepatoma cells were treated as in Figure 4A for 48 h and the proteins were isolated and analyzed by western blotting. GAPDH expression served as an internal control. **(D)** Flow-cytometric analysis of apoptosis was performed after treatments with sorafenib and/or auranofin. **(E)** Quantification of cell death detected in Figure 4C. Cell apoptosis as detected by PI staining. Values represent the mean percentage of dead cells ± SD from three samples (*P < 0.05,***P < 0.001). **(F)** Schematic illustration showing the PCK1–NADPH–ROS-TXNRD1 axis. In HCC, decreased PCK1 suppressed the generation of NADPH via pentose phosphate pathway (PPP). Subsequently, ROS, which is quenched by NADPH, was increased, further promoted nuclear translocation of Nrf2 and the expression of TXNRD1. The cropped blots are used in the figure and full length blots are presented in Figure S6.

To explore a potential treatment strategy for HCC, we tested the apoptosis-inducing effect of sorafenib (the first drug approved for advanced HCC treatment) in combination with auranofin (an inhibitor of thioredoxin reductase) *in vitro*. PCK1-KO PLC/PRF/5 cells were treated with 0.6 μmol/L auranofin or 5 μmol/L sorafenib, alone or in combination. The cell proliferation rate was increased in PCK1-KO cells as compared to parental cells. Furthermore, combined treatment with sorafenib and auranofin induced a synergistic inhibitory effect on cell proliferation in PCK1-KO cells (Figure 5A). In addition, excess ROS accumulation were observed in PCK1-KO cells treated with auranofin plus sorafenib (Figure 5B). Apoptosis is one of the most important factors affecting cell proliferation. To investigate the effects of sorafenib and auranofin on apoptosis, we detected the protein levels of Caspase3/9, Cleaved caspase 3/9, Bax and Bcl-2, and analyzed the Bax/Bcl-2 ratio, which indicates apoptosis occurrence. PCK1-KO cells treated with sorafenib exhibited increased Cleaved caspase3, Cleaved caspase9 and Bax levels, while cells treated with auranofin plus sorafenib showed significantly increased these proteins expression as compared to cells treated with sorafenib alone. The Bax/Bcl-2 ratio was markedly increased in the PCK1-KO cell exposed to combination treatment as compared to parental cells or each PCK1-KO exposed to single treatments. Furthermore, the protein levels of TXNRD1 were decreased in cells treated with auranofin plus sorafenib, compared to those in PCK1-KO cells treated with auranofin alone (Figure 5C). Flow-cytometric analysis revealed that treatment with auranofin or sorafenib induced cell apoptosis. Cells treated with auranofin plus sorafenib showed significantly higher apoptosis (*P* < 0.001; Figures 5D,E). Taken together, these *in vitro* data suggested auranofin could increase the sensitivity of PCK1-KO cells to sorafenib.

## Discussion

Metabolic reprogramming is a hallmark of cancer, plays an important role in tumor proliferation, metastasis and drug-resistance by providing essential cellular components such as energy equivalents, redox cofactors and biomass building blocks. (24–27) Our results demonstrated that PCK1 was significantly decreased in HCC tumor tissues compared with adjacent normal tissues, which was verified by analysis of data obtained from the TCGA database. Furthermore, lower PCK1 expression correlated with poorer prognosis. Consistent with previous studies (4, 9), our data suggest a tumor-suppressor function of PCK1 in HCC.

However, elevated PCK1 expression has also been found in other types of cancers, such as melanoma (8), breast cancer (26), and colorectal cancer (7), where it supports anabolic pathways and cell proliferation. The distinct expression profile of PCK1 in HCC tissues compared to other solid tumors can be explained by the fact that in the liver, which accounts for approximately 90% of gluconeogenesis, the upregulation of gluconeogenesis by PCK1 results in the suppression of hepatoma proliferation. Specifically, PCK1-induced promotion of gluconeogenesis enhances energy consumption and attenuates energy production, including glycolysis, and mitochondrial respiration, which results in ATP depletion. Decreased aerobic glycolysis and mitochondrial respiration pathway also suppresses the generation of metabolic intermediates as building blocks for cells (28). Forced PCK1 expression in glucose-starved hepatoma cells reportedly induces tricarboxylic acid cycle cataplerosis, leading to energy crisis, oxidative stress, and cell apoptosis (4).

In cancer cells, excess ROS are generated through increased anabolism, decreased catabolism, or mitochondrial dysfunction. Previous studies have shown that glucose metabolism-related enzymes, such as PKM and FBP1 (29–31), can participate in the regulation of ROS production. In our study, low expression of PCK1 resulted in elevated intracellular ROS level *in vitro* and *in vivo*. PCK1 plays a crucial role in hepatic anabolism and catabolism (4, 22, 32). Thus, PCK1 is likely to regulate the ROS level through altering the metabolism of HCC. Ko et al. reported that PCK1 deletion in myeloid cells increases the intracellular ROS level (33). Ma et al. reported that 3-MPA, an inhibitor of PCK1, could increase ROS in CD8^+^ memory T cells through the abrogation of the PCK1-glycogen-pentose phosphate pathway (PPP) (34). Since NADPH is a key product of PPP, and plays an essential role in the ROS generation via glutathione (GSH) system, we hypothesized that glucose-6-phosphate which indirectly catalyzed by PCK1 is channeled to PPP. Our results demonstrate that PCK1 overexpression increases the ratio of NADPH/NADP^+^, and further decreases ROS production. However, it has been reported that forced PCK1 expression in glucose-starved HCC cells induces a high ROS level (4). Thus, the specific mechanism of PCK1 affecting ROS through metabolic regulation under different culture conditions requires further investigation.

Our data further showed that overexpression of PCK1 abrogated the nuclear translocation of Nrf2 and the expression of its downstream gene TXNRD1. The transcription factor Nrf2 is considered a major transcriptional regulator in the defense against ROS (35, 36). TXNRD1 is a critical antioxidant enzyme that catalyzes the NADPH-dependent reduction of thioredoxin to regulate cellular redox homeostasis (37, 38). TXNRD1 is upregulated in many human malignancies, and inhibition of the TXN pathway causes hepatoma cell death (17, 39–41). Furthermore, auranofin, a TXNRD1 inhibitor, has been reported to induce apoptosis and to enhance the anticancer activities of chemotherapeutic agents (16, 42, 43). In line herewith, we observed that TXNRD1 may be related to drug resistance of hepatoma cells. Sorafenib, a multi-kinase inhibitor, is the first-line therapy for patients with advanced HCC (1). However, considering the sorafenib resistance crisis in HCC, the development of new combination therapies with sorafenib is urgently required. Recent studies reported that synergistic combination of sorafenib and uracil-tegafur or octreotide can improve the sensitivity toward sorafenib in the treatment of HCC (44, 45). This study confirmed that sorafenib in combination with auranofin exhibits a stronger pro-apoptotic effect *in vitro*.

In conclusion, our study demonstrated that PCK1 is significantly downregulated in HCC, and PCK1 deficiency decreases NADPH/NADP+ ratio, increases cellular ROS levels, which results in activation of Nrf2 and expression of TXNRD1(Figure 5F). In addition, auranofin enhances the sensitivity to sorafenib via inhibition of TXNRD1 in PCK1-knockout cells *in vitro*. Our finding provides new insights into the mechanism of antioxidant enzyme gene TXNRD1 mediated by altered metabolism in liver cancer, and might offers a potential therapy for HCC.

## Ethics Statement

All patients provided informed consent, and this study was conducted with the approval of Institutional Ethical Review Board of Chongqing Medical University (project license number: 2017012). All animal experiments were carried out according to the guidelines of the Institutional Animal Care and Use Committee at Chongqing Medical University (The project license number: 2017012), and protocols of animal care and use adhere to National Regulations for the administration of laboratory animals.

## Author Contributions

KW and NT conceived and designed the study. LT, JX, XP, QG, GZ, and YY performed experimental work. LL and JX performed data analysis. LT prepared the manuscript. KW and NT provided administrative support and funded experiments. All authors read and approved the final manuscript.

### Conflict of Interest Statement

The authors declare that the research was conducted in the absence of any commercial or financial relationships that could be construed as a potential conflict of interest.
