# Supplementary figures and images for: PCK1 Downregulation Promotes TXNRD1 Expression and Hepatoma Cell Growth via the Nrf2/Keap1 Pathway
Source: Front Oncol. 2018 Dec 17;8:611. doi: 10.3389/fonc.2018.00611 (PMC6304441; doi:10.3389/fonc.2018.00611)

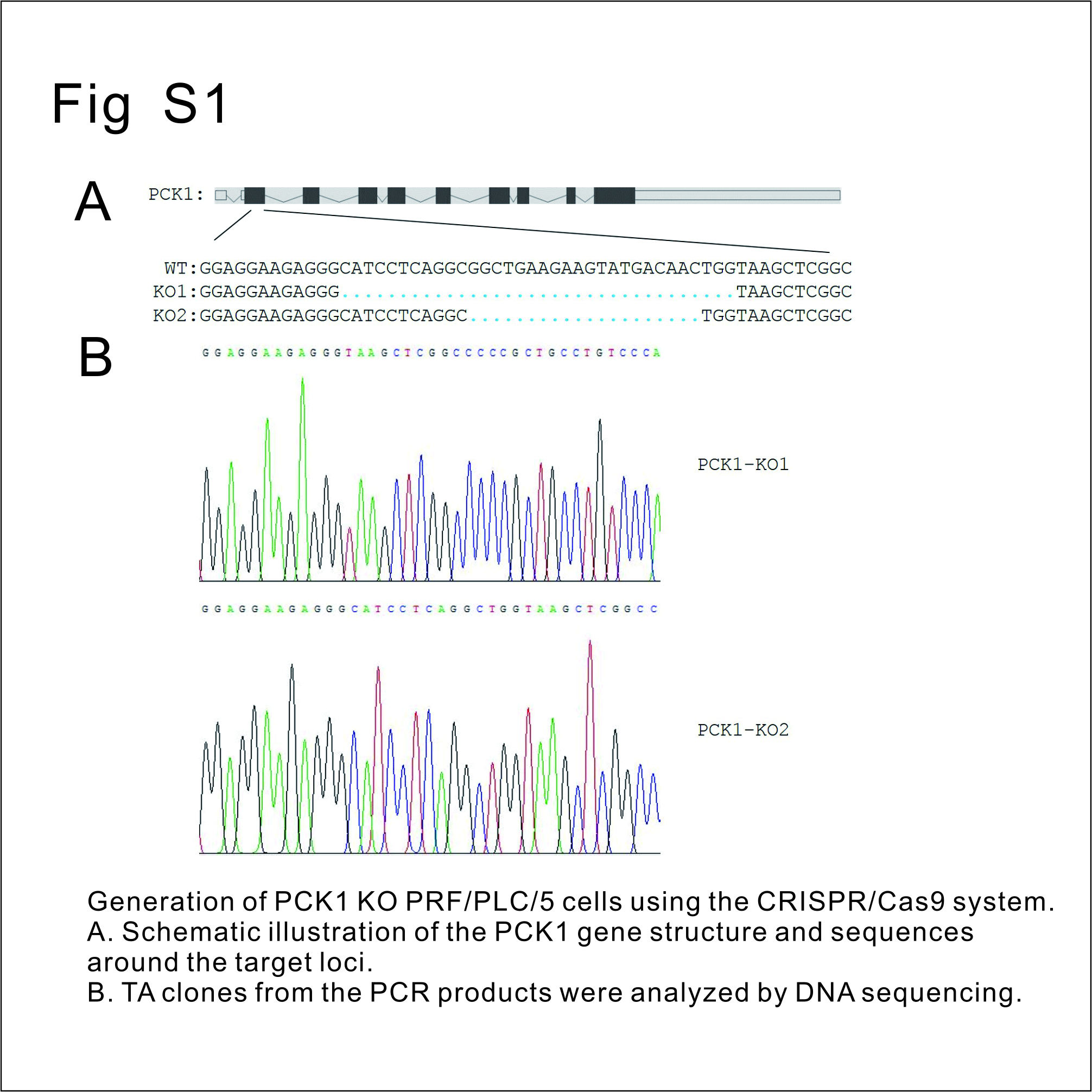

Supplement: Supplementary file 1 [file Image_1.JPEG]

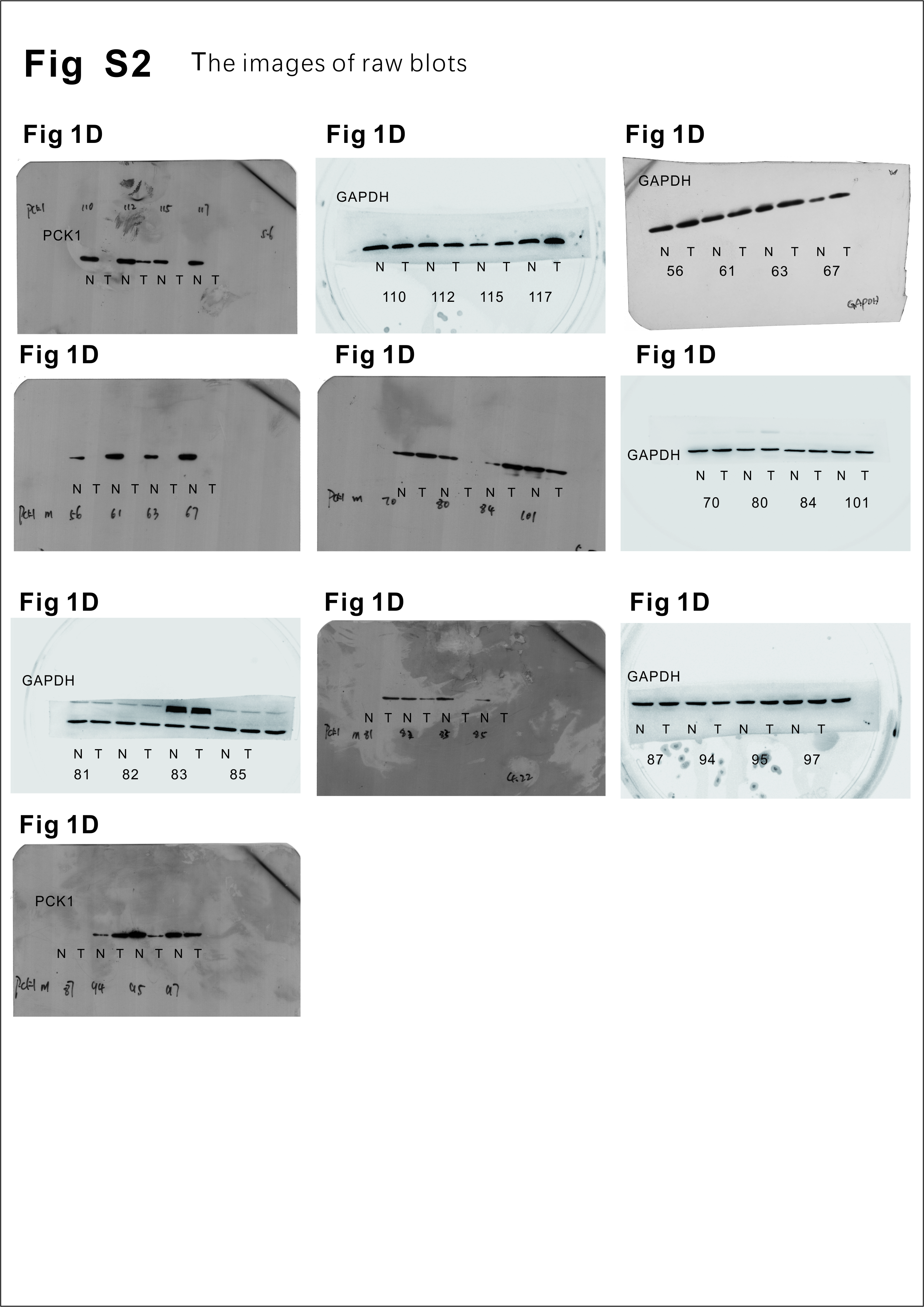

Supplement: Supplementary file 2 [file Image_2.JPEG]

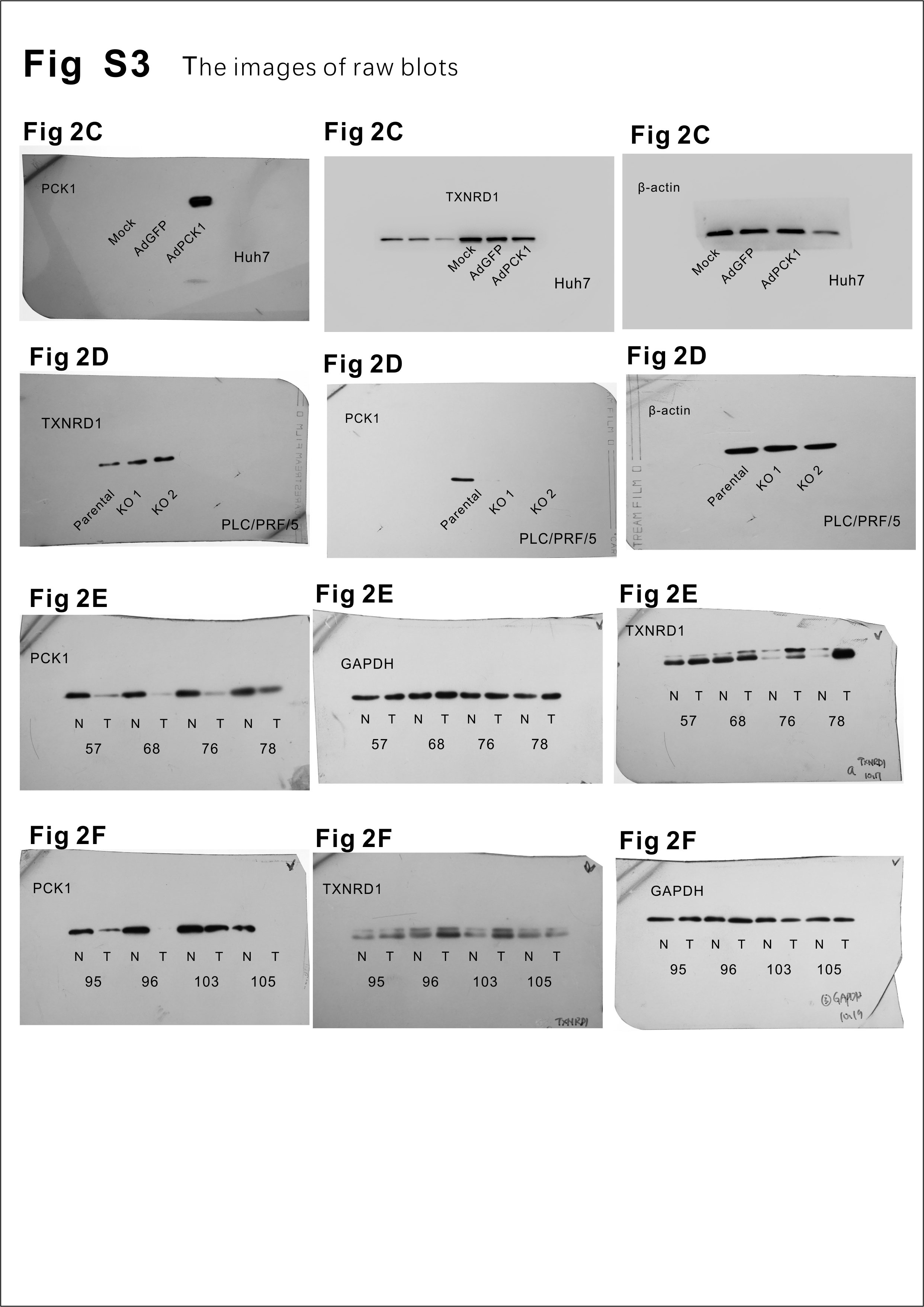

Supplement: Supplementary file 3 [file Image_3.JPEG]

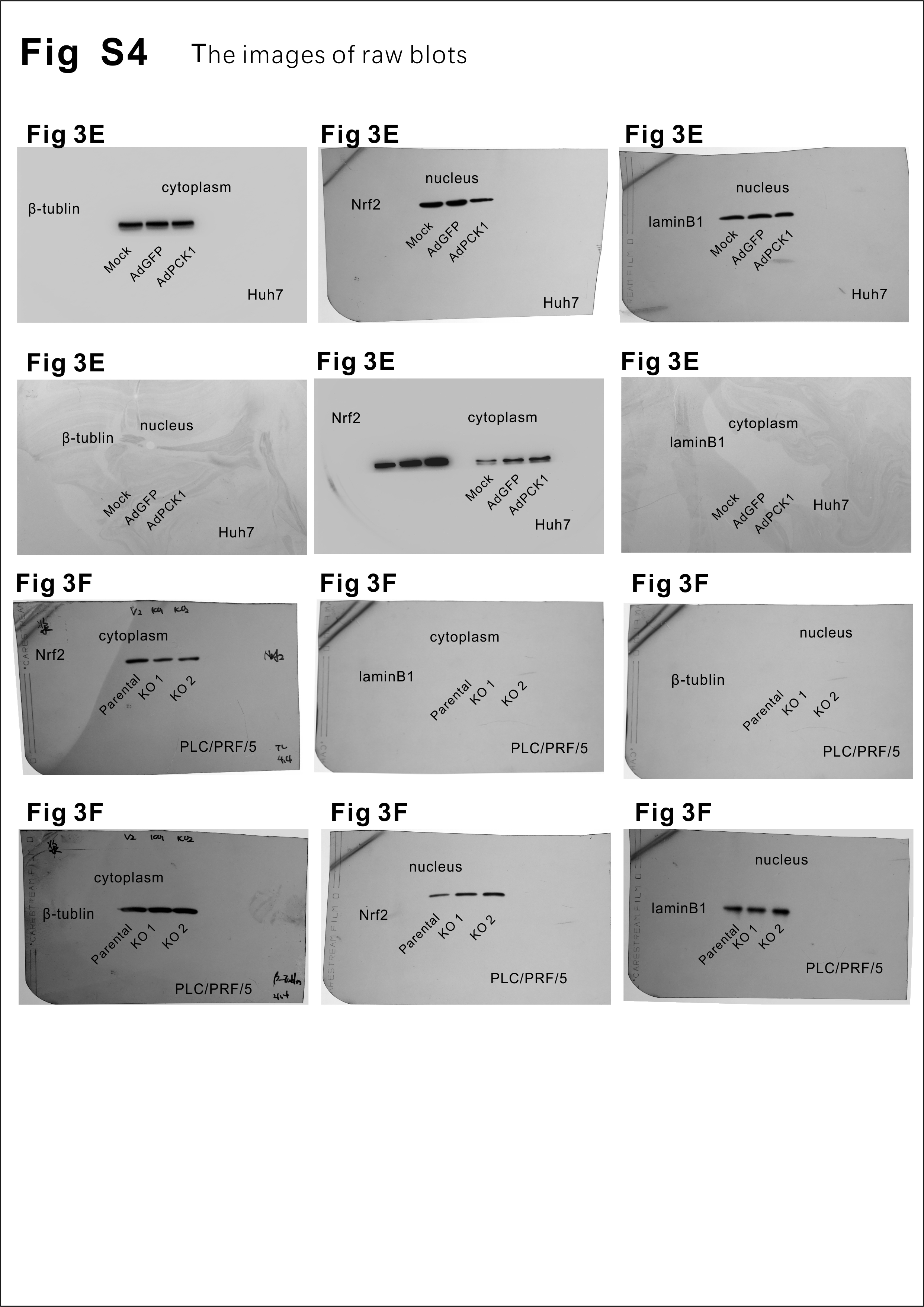

Supplement: Supplementary file 4 [file Image_4.JPEG]

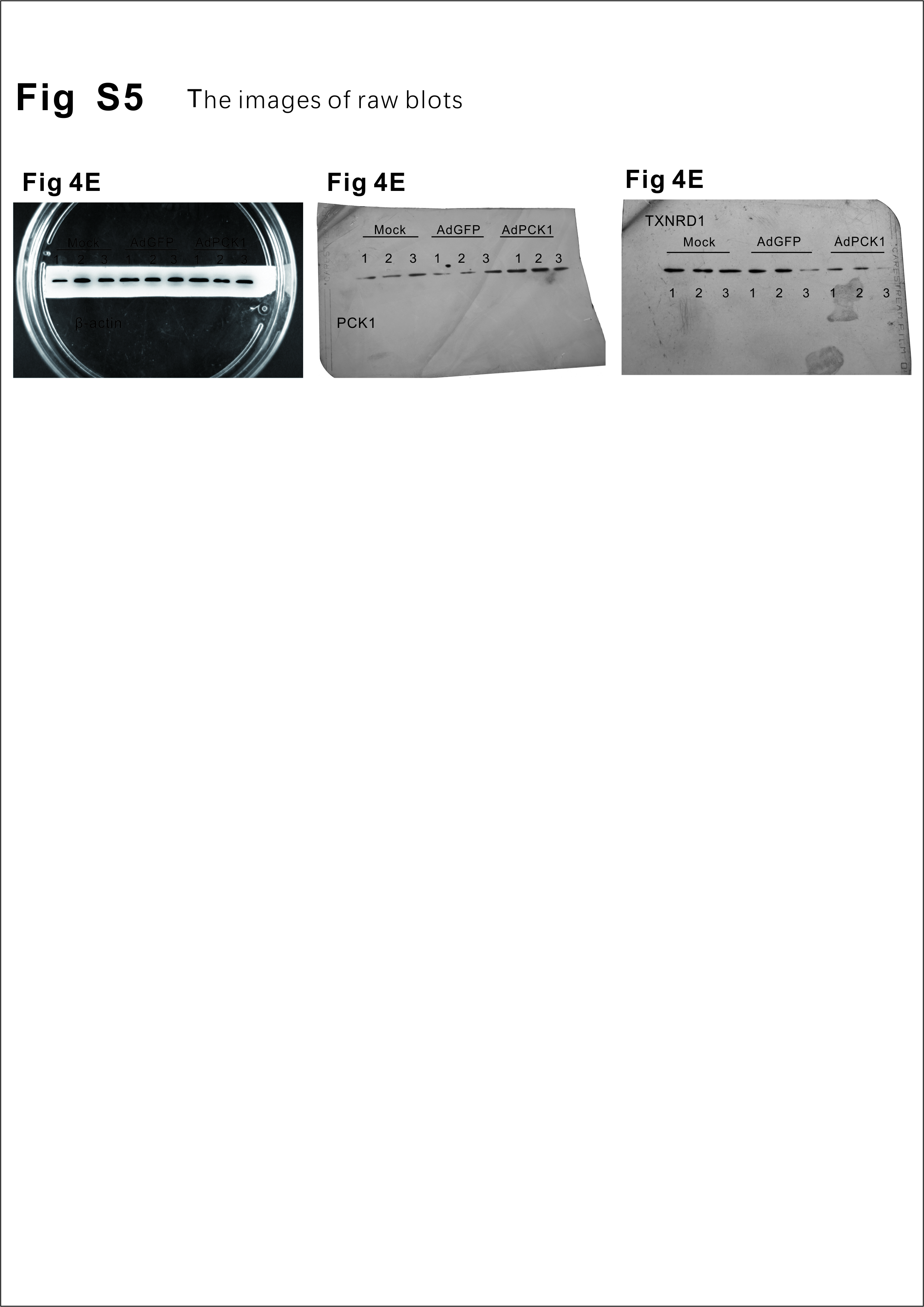

Supplement: Supplementary file 5 [file Image_5.JPEG]

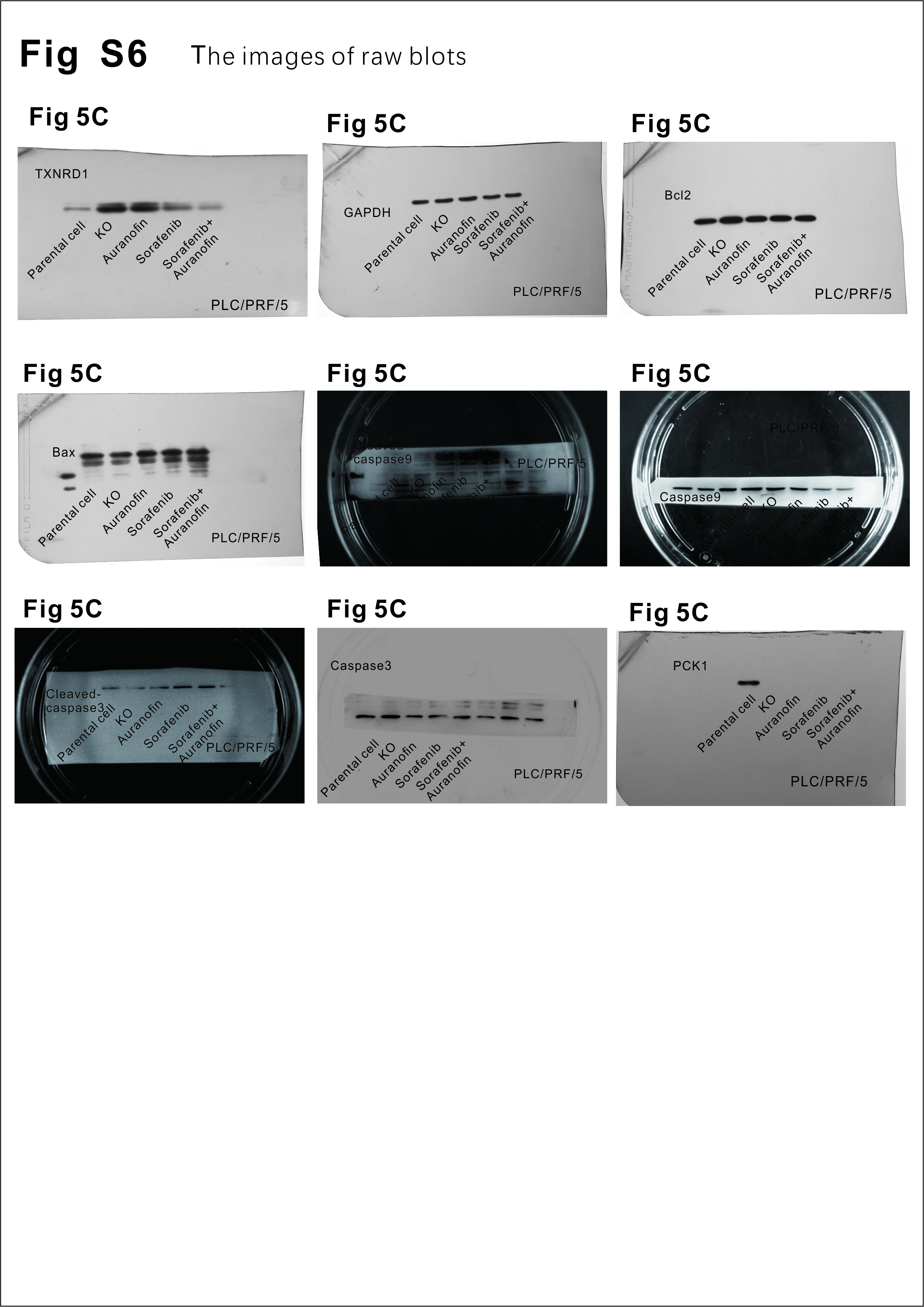

Supplement: Supplementary file 6 [file Image_6.JPEG]
